# An in silico evaluation of signal and separability properties of k-edge materials in spectral CT

**DOI:** 10.1038/s41598-025-21693-0

**Published:** 2026-02-05

**Authors:** Jayasai R. Rajagopal, Faraz Farhadi, Pooyan Sahbaee, Elizabeth C. Jones, Ehsan Samei, William F. Pritchard

**Affiliations:** 1 Carl E. Ravin Advanced Imaging Laboratories and Center for Virtual Imaging Trials, Department of Radiology, Duke University Medical Center, Durham, NC 27705 USA; 2 Radiology and Imaging Sciences, National Institutes of Health Clinical Center, Bethesda, MD 20892 USA; 3 Siemens Healthineers, Malvern, PA 19335 USA

**Keywords:** Translational research, Computed tomography

## Abstract

Spectral CT can acquire signal at multiple x-ray energy levels. This enables material quantification by exploiting differences in x-ray attenuation across energy levels, particularly for k-edge materials. This simulation study quantified the signal and separability of current and potential clinical contrast agents across a range of materials and energies. A validated CT simulation platform was used to simulate a clinical photon-counting CT scanner with two energy thresholds. A cylindrical phantom containing common biological materials, clinical contrast agents, candidate contrast agents and nanoparticles, and investigational materials was imaged with varying upper energy thresholds (50–90 keV). At each energy level, images were assessed for noise, each material was assessed for contrast, and each material pair was evaluated for separability. Material contrasts reached peak value at the closest threshold higher than their respective k-edge. The energy threshold that produced the highest separability for each pair was characterized. Selection of energy threshold was dependent on the materials of interest. Threshold values at or just above a material’s k-edge maximized material signal while separability was maximized by the threshold that best separated k-edge signals.

## Introduction

Since its invention in the 1970s, x-ray based computed tomography (CT) has been a premier imaging modality for diagnostic imaging. The number of CT scans^1,2^ has increased over time across the globe. CT plays an important role in diagnosis for a variety of areas including thoracic, abdominal, cardiac, neurological, musculoskeletal, oncologic, and emergency care. This role can be attributed to the many benefits of CT including high spatial resolution, volumetric imaging, tissue contrast, and short scan time^3^.

The mechanism that enables computed tomography is photon attenuation. Attenuation^4^ describes the loss of flux as a photon beam passes through any object. Attenuation is determined by the length of material that photons pass through, the composition of those materials, and the energy of the photon. CT exploits this behavior to discriminate between tissues with subtly different radiographic contrast, that is, differences in x-ray attenuation. To improve differentiation of different soft tissues, which have similar material compositions, contrast agents can be used to improve the attenuation of target tissues. The most commonly used contrast agent in clinical CT is iodine. The addition of a contrast agents enables a number of applications including organ and tissue characterization, angiography, perfusion studies, and tumor differentiation.

Conventional computed tomography relies on an integrated x-ray energy spectrum for material discrimination. Spectral CT^5,6^ represents an extension of conventional CT by acquiring spectral CT image data simultaneously or near simultaneously at multiple energy levels. Clinical systems primarily use dual-energy implementations^7^, which use a variety of techniques^8^ to acquire two energy levels at once. More recently, photon-counting CT (PCCT) has offered a new method to obtain spectral information using a different detection method than conventional CT, enabling acquisition of images at more than two energy levels^9,10^ and processing that information in a variety of post-processing options^11^. Some post-processing options generate images that only contain specific portions of acquired signal, such as virtual non-contrast images^12,13^ and virtual monoenergetic images^14,15^. Other methods enable domain transformation from signal intensity. Examples include rho-effective Z^16,17^, photoelectric-Compton scatter^18^, and basis material decomposition^19^.

Material decomposition is a technique that uses signal acquired at multiple energies to quantify the location and amount of materials. Material specific characterization is possible as materials have different attenuations at different x-ray energy levels. This technique is particularly potent when taking advantage of the k-edge effect. The k-edge effect is caused by a discontinuous increase in signal at certain energy levels due to the photo-electric effect, the interaction of x-ray photons at or above the binding energy of an atoms k-shell electrons^20–22^. Different materials have different k-edge levels, including iodine at 33.2 keV. Material decomposition applications can be further enhanced when considering multiple k-edge materials used in concert to target different anatomies or functions. Pre-clinical investigations have studied multi-phase multi-contrast applications^23^ and using multiple contrast agents for different anatomical regions^24,25^.

While iodine is the primary clinical contrast agent, there are many elements with k-edge energies in the range of diagnostic x-rays that are promising candidates for k-edge imaging^26^. Both gadolinium^27,28^ and bismuth^29^ have been investigated as contrast agents in CT. As an alternative to liquid contrast agents, studies have also focused on several nanoparticle agents^30–32^ and on drug eluting beads^33,34^ for therapeutic applications. The introduction of new contrast agents increases the capabilities of CT to include multi-contrast agent studies^23,24,35^ and functional imaging applications^36,37^.

While earlier investigations of candidate materials have focused on identifying viable contrast agents and optimization of tube voltage for imaging an agent^38,39^, there are some challenges to pursuing further experimental comparisons. Acquisition and synthesis of required material compounds can be difficult and requires specialized equipment or expertise. Further, inhomogeneities within solutions can complicate validation of the imaged ground truth. As an alternative, virtual imaging trials^40–42^ can address these challenges. Physics-based simulations can accurately reflect reality while reducing cost and complexity into the computational domain and providing a known ground truth.

One important consideration with potential new contrast agents is how those materials affect signal in spectral CT acquisitions. For clinical applications, the knowledge of acquisition parameters is important to maximize signal and to produce the best clinical outcomes. Further, as spectral CT becomes more integrated in the clinic, there is an increased opportunity for multi-contrast agent imaging. The relationship between pairs of k-edge materials and their signal responses under different energy separation conditions has been underreported. The goal of this work was to characterize the signal and separability properties of materials in relation to photon-counting CT energy thresholds and provide a framework for future development. This was done by applying an established spectral metrology to simulated data using a validated simulation platform, DukeSim^43^. Materials used in this study included common biological elements, current contrast agents, and candidate k-edge materials. The dependence of contrast properties and material separability was characterized as a function of energy threshold.

## Results

### Virtual Phantom design

**Fig. 1:**
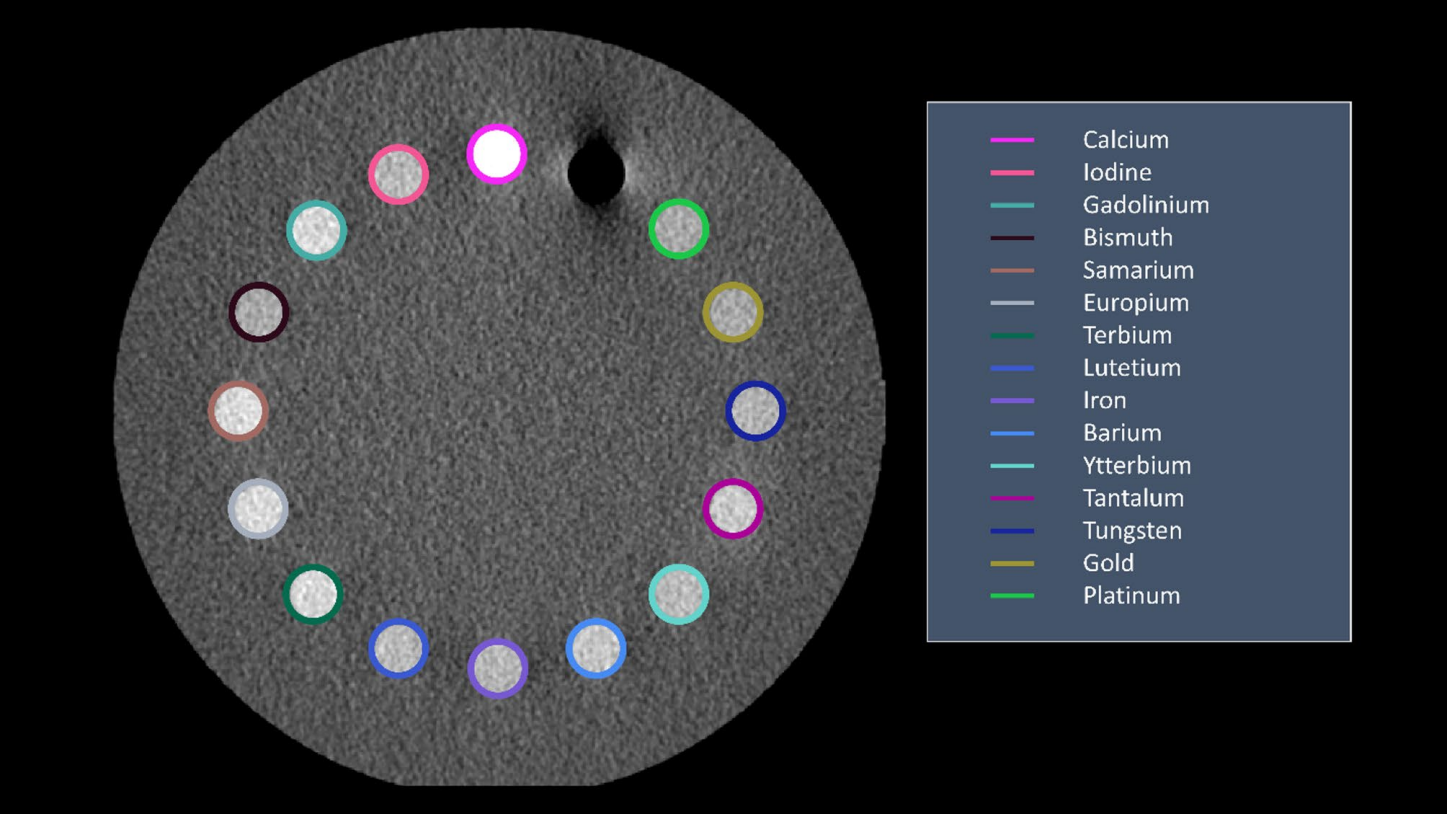
Low energy threshold (20–50 keV) image of cylindrical computational phantom. The colored rings identify the different materials of interest. Background is water. Image presented with window width/window level of 300/40.

This study used a cylindrical computational (30 cm) phantom containing 16 inserts (Fig. 1). The background of the phantom was filled with water and inserts were filled with materials of interest at fixed concentrations. Materials were chosen to represent elements found in the body (calcium, iron), currently used contrast agents (iodine, barium ), candidate contrast agents (gadolinium^27,44^, bismuth^29^, candidate nanoparticle materials (ytterbium^45,46^, tantalum^47,48^, tungsten^49^, gold^50,51^, platinum^52^, and lanthanides that did not fall into the other groups (samarium, europium, terbium, lutetium). Table 1 provides details of each material including their k-edge and reason for inclusion. Material concentrations were matched to iodine for contrast agents and nanomaterials. Calcium and iron concentrations were chosen to match the lowest concentration of inserts in commercial dual-energy phantoms.

**Table 1:** Elements used in this study, including their k-edges, the concentrations used, and the reason for inclusion.

Element	k-edge (keV)	Concentration	Reason for inclusion
Calcium (Ca)	4	50 mg/mL	Commonly found in the body
Iron (Fe)	7	30 mg/mL	Commonly found in the body
Iodine (I)	33	4 mg/mL	Currently used contrast agent
Barium (Ba)	37	4 mg/mL	Currently used as a contrast agent
Samarium (Sm)	47	4 mg/mL	Investigational usage
Europium (Eu)	49	4 mg/mL	Investigational usage
Gadolinium (Gd)	50	4 mg/mL	Currently used contrast agent for MRI and candidate agent for CT
Terbium (Tb)	52	4 mg/mL	Investigational usage
Ytterbium (Yb)	61	4 mg/mL	Candidate nanoparticle agent
Lutetium (Lu)	63	4 mg/mL	Investigational usage
Tantalum (Ta)	67	4 mg/mL	Candidate nanoparticle agent
Tungsten (W)	70	4 mg/mL	Candidate nanoparticle agent
Platinum (Pt)	78	4 mg/mL	Candidate nanoparticle agent
Gold (Au)	81	4 mg/mL	Candidate nanoparticle agent
Bismuth (Bi)	91	4 mg/mL	Candidate contrast agent

For further evaluations, materials were grouped into three categories based on function and range of k-edge values: common biological materials and current clinical contrast agents (barium, calcium, gadolinium, iron, iodine), lanthanides (europium, gadolinium, lutetium, samarium, terbium), and candidate contrast agents and nanomaterials (bismuth, gold, platinum, tantalum, tungsten, ytterbium).

### Image quality

Image quality metrics were evaluated to gain an understanding of the relationship between material signal and energy thresholds. Regions of interest (ROIs) were drawn in each insert and water background. Water background ROIs were used to calculate noise which represents the uncertainty associated with each signal condition. Contrast was evaluated as the difference between signal in each insert and background which represents the detected signal for each material at each energy threshold.

**Fig. 2:**
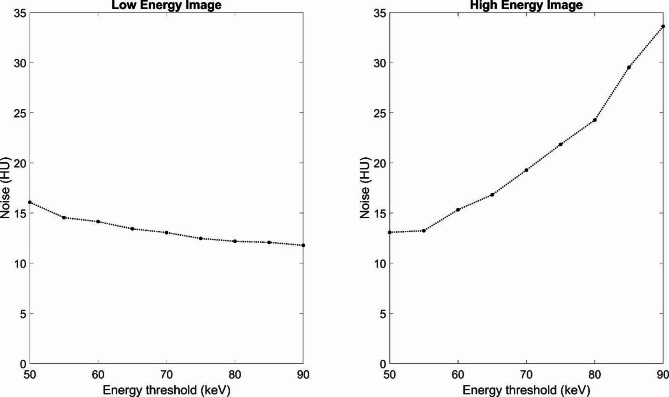
Image noise (y-axis) as a function of energy threshold (x-axis). Noise was measured in a central water background region of interest.

As the upper energy threshold was increased from 50 to 90 keV, the noise in the lower energy image fell from 16.0 to 11.8 HU while it increased in the high energy image from 13.0 to 33.6 HU (Fig. 2).

**Fig. 3:**
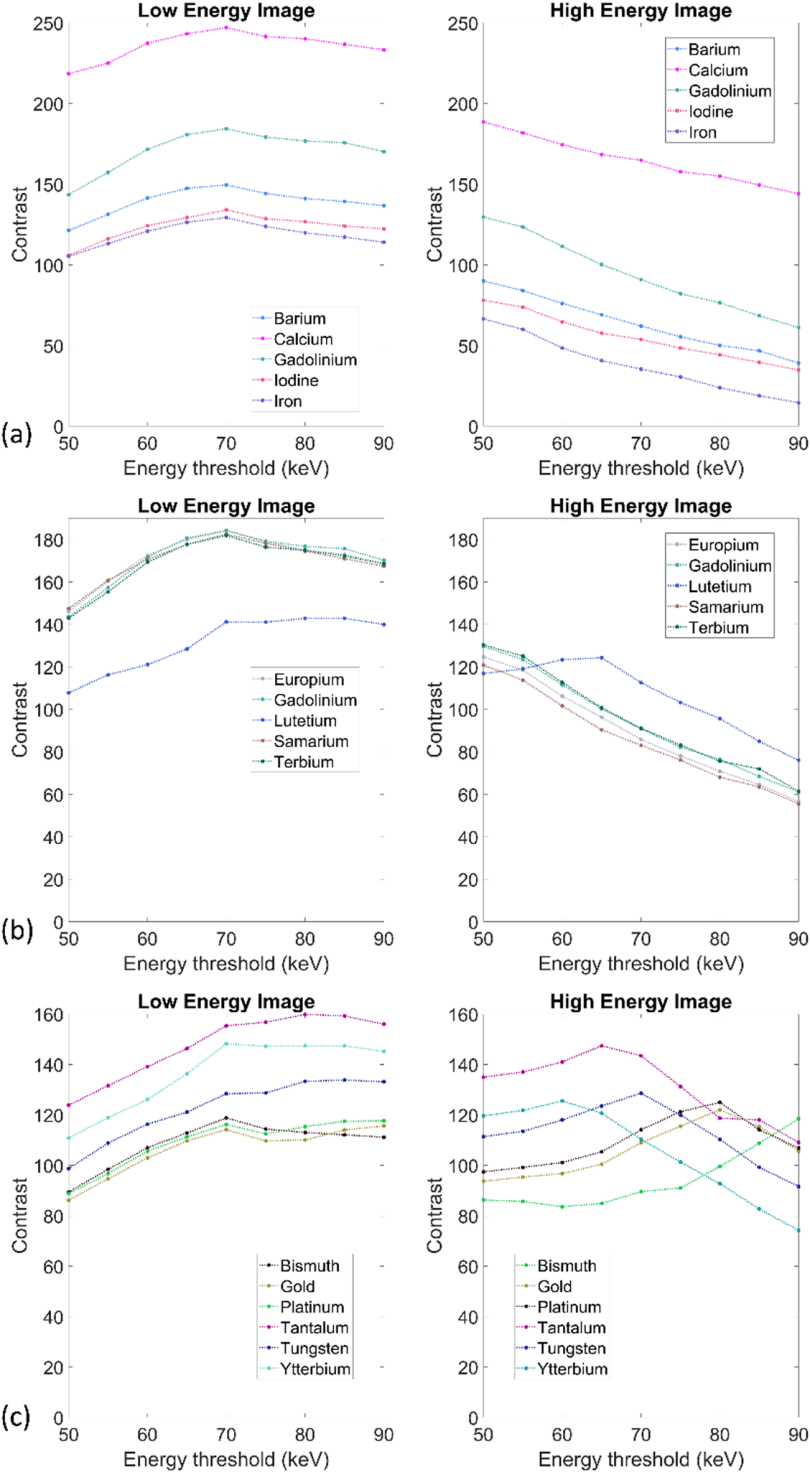
(**a**) Contrast (y-axis) as a function of energy threshold (x-axis) for common biological materials and currently used contrast agents. Low energy image on the left and high energy image on the right. (b) Contrast (y-axis) as a function of energy threshold (x-axis) for lanthanides. Low energy image on the left and high energy image on the right. (**c**) Contrast (y-axis) as a function of energy threshold (x-axis) for candidate nanomaterials and contrast agents. Low energy image on the left and high energy image on the right.

For the first group of materials (Fig. 3a), containing common biological materials and current clinical contrast agents, contrast in the lower energy image increased with energy threshold before peaking at 70 keV. For the higher energy image, contrast fell as the energy threshold was increased. Calcium, the material with the highest concentration in the dataset, had the highest contrast across all thresholds and images.

For the second group of materials (Fig. 3b), containing the lanthanides, contrast in the lower energy image increased steadily with energy threshold and began to plateau after 70 keV. In the higher energy image, contrast decreased as energy threshold increased for all materials except lutetium which instead peaked at 65 keV. Across all the lanthanides included in this dataset, lutetium had the lowest contrast in the low energy image for each threshold but had the highest contrast in the high energy image for every energy threshold greater than 60 keV.

For the third group of materials (Fig. 3c), containing high-Z candidate contrast agents and nanomaterials, contrast increased with energy threshold in the low energy image for tantalum, tungsten, and ytterbium. Bismuth, gold, platinum, and ytterbium reached a peak value at 70 keV after which the contrast decreased. For the high energy image, the contrast of tantalum, tungsten, and ytterbium reached peak values between 60 and 70 keV before decreasing. Gold and platinum increased until reaching peak values at 80 keV. The contrast of bismuth continued to increase up to 90 keV.

### Separability

Separability between two materials was calculated using a previously-described technique^53^ in terms of the separability index. This method calculates the expected overlap in signal component between two materials of interest. A technique or threshold choice that offers higher separability between two materials would offer a higher chance to distinguish those two materials minimizing confusion between the two.

**Fig. 4:**
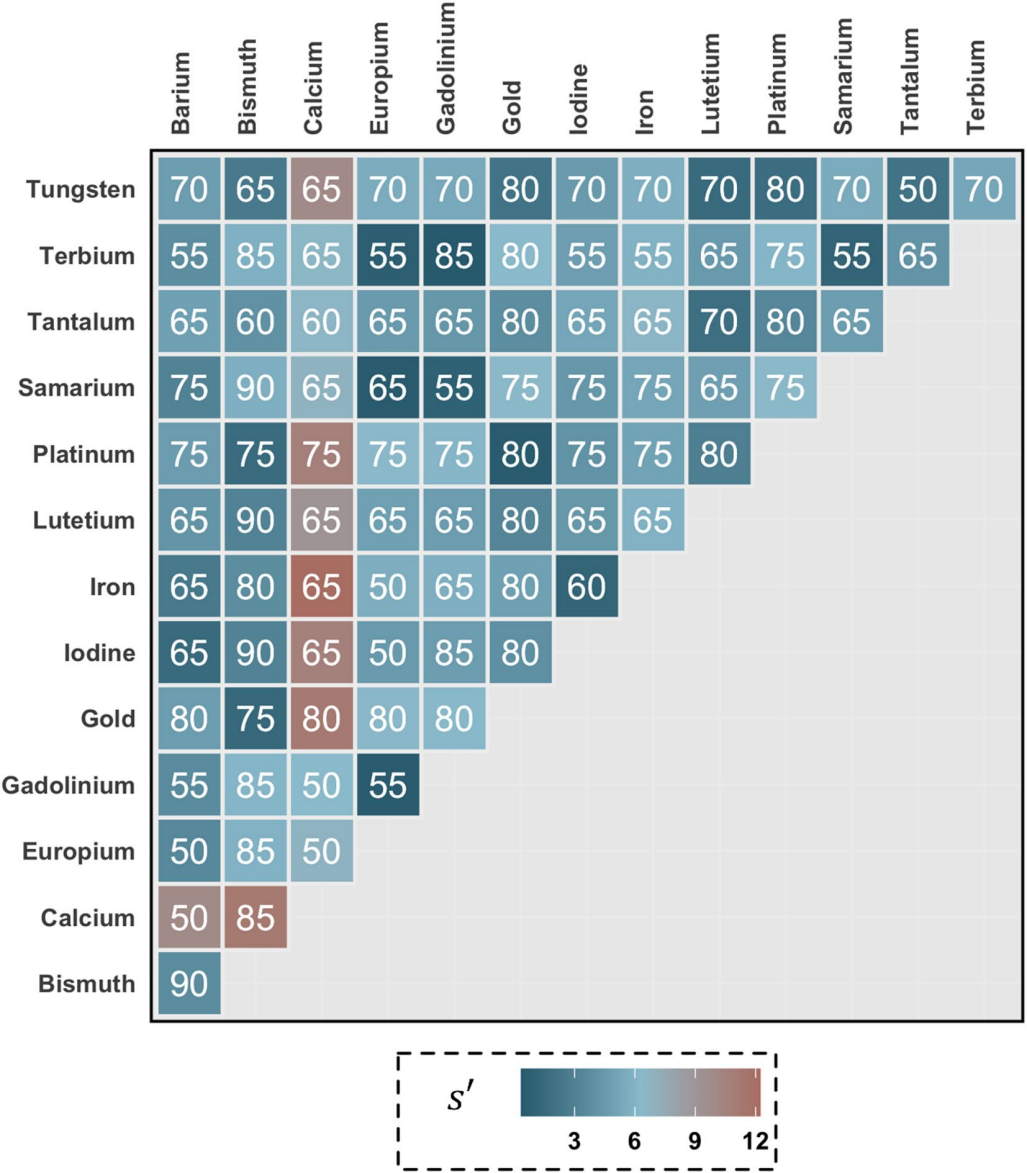
High energy image threshold values (keV) that generate the highest separability for each pair of materials. Separability (s’) between pairs of materials is illustrated by the color shading of each cell. The numbers within each cell indicate the high energy threshold value that provided the most separation between those two materials.

**Fig. 5:**
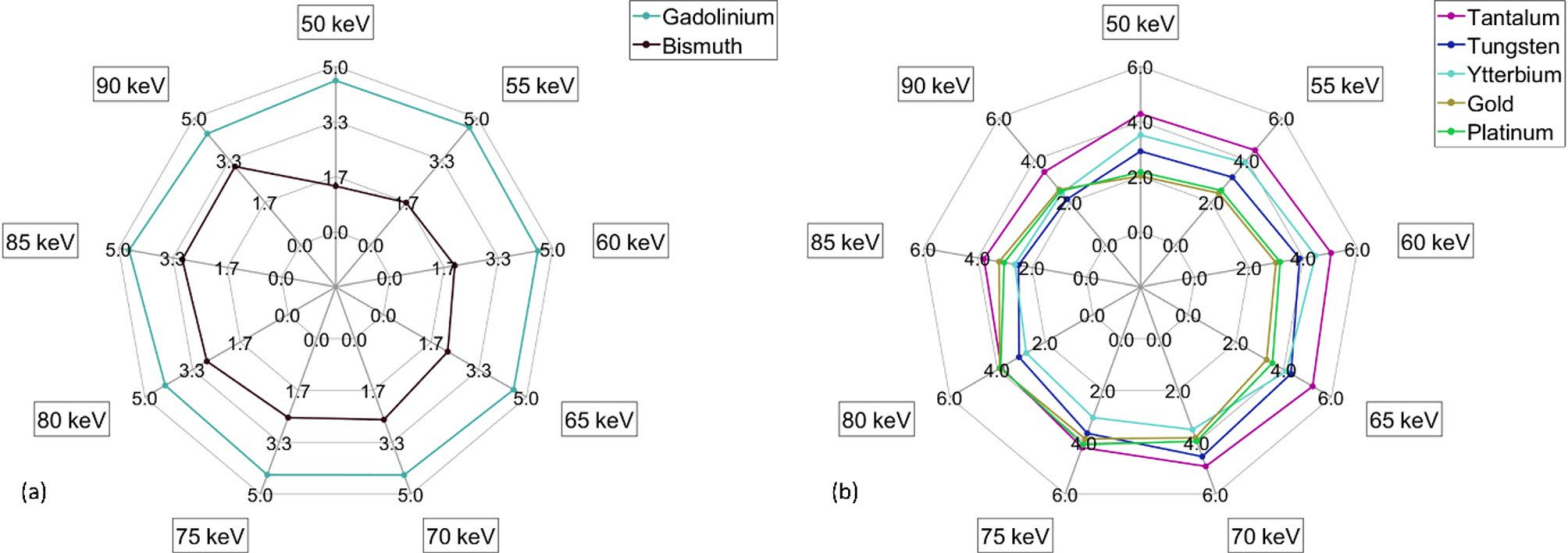
Separability of iodine and potential dual contrast agents. (**a**) Spider plot showing the separability of gadolinium and bismuth with iodine. (**b**) Spider plot showing the separability of candidate nanomaterials with iodine.

The high energy image threshold value that generated the peak separability for each pair of materials is shown (Fig. 4). Separability of gadolinium and bismuth with iodine is of particular interest (Fig. 5a). The iodine/gadolinium pair maintained a comparable level of separability across all thresholds. The separability of the iodine/bismuth pair steadily increased with energy threshold.

Separability between iodine and each of the nanomaterial agents increased until it reached a peak at the threshold closest to that material’s k-edge and steadily decreased from that point (Fig. 5b). Tantalum had the highest separability at all thresholds except 80 keV where both platinum and gold had a comparable value.

**Fig. 6:**
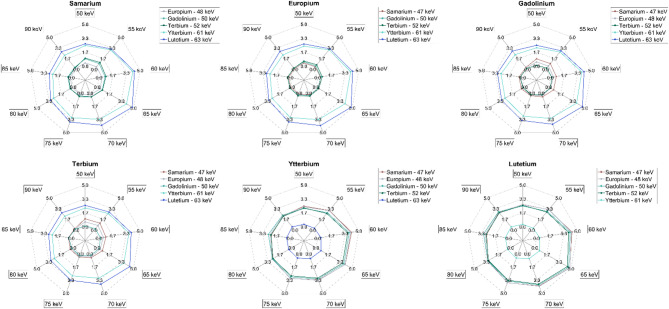
Spider plots showing the separability of each lanthanide with the other lanthanide agents. Plots ordered by atomic number and k-edge: (**a**) Samarium, (**b**) Europium, (**c**) Gadolinium, (**d**) Terbium, (**e**) Ytterbium, (**f**) Lutetium.

When comparing their separability to one another (Fig. 6), the lanthanides that had k-edge’s close to one another had low separability. Higher thresholds caused the lowest separability for all combinations of lanthanides while peak separability was between 55 and 65 keV depending on the lanthanide pair.

## Discussion

The primary goal of this study was to characterize signal and separability properties of materials in spectral CT in relation to the energy threshold. This was accomplished via simulation of a clinical photon-counting CT system to image a cylindrical water phantom containing materials of interest. We found that for specific materials, signal peaked with an energy threshold value just above that material’s k-edge, and that for pairs of materials, the highest separability was maximized by the threshold that best separated the k-edges of those materials.

As the focus of this study was on change in signal characteristics due to the interaction of material properties and specific acquisition parameters, the simulated setup was chosen to minimize signal variations due to other external factors. By using a simple cylindrical phantom with a fixed size and water background, several complex effects were simplified in our signal calculations. First, the size of the phantom influences detected signal due to photon starvation. A larger phantom size requires a longer path-length for each individual photon which results in fewer photons reaching the detector and reduces the overall quality of signal^54,55^. We would expect a direct relationship between phantom size and the reported signals and separabilities in this study. This effect would be particularly magnified for those materials with a lower k-edge as low energy photons are more susceptible to beam hardening. Second, a tissue background that more realistically reflected the complexity and heterogeneity of human patients would introduce further uncertainty. Accounting for different tissue types, structural noise variations, and textures would further obscure the targeted signal. Consideration of these more complex scenarios using size variant and anthropomorphic human phantoms will be considered in future studies.

From the image quality analysis, the most important finding was the relationship between noise and the upper energy threshold. As the threshold was increased fewer photons were used to form the high energy image causing noise in the higher energy image to rapidly increase. For materials, such as platinum, gold, and bismuth, where a higher value for the upper threshold maximized signal or separation from other materials, the impact of noise needs to be carefully monitored. This is particularly relevant for any post-processing applications, such as material decomposition, where noise in acquired images rapidly propagates in the post-processing workflow^56,57^. Measures to mitigate the noise deficit can include hardware changes to increase overall signal, such as increasing tube voltage or current, and software corrections, including reconstruction parameters and noise correction.

Another relevant finding from the image quality analysis was the impact of the k-edge effect. Across all material groups, the signal characterization clearly demonstrated the k-edge effect based on the location of the signal peaks. For several materials, particularly in group two, the contrast peak occurred closer to 70 keV than a lower value as would be expected which may be attributed to the combination of the k-edge effect and the overall shape of the x-ray spectrum. The findings from this section of the study can help inform the selection of energy thresholds when considering single contrast material scenarios. Proper selection of energy thresholds can extract maximum signal while minimizing the impact of noise when a single contrast material is used.

Separability between materials is an important precondition for material decomposition as identification and quantification relies on distinguishable signals. In this work, this was characterized using the separability index. When comparing two sets of imaging parameters for a pair of materials, the parameters resulting in a higher separability index indicate that the materials in that pair are easier to differentiate than parameters with lower separability. The separability index accounts for the impact on signal overlap due to image formation factors including material concentration and reconstruction conditions. Notably, small differences in attenuation due to similar material concentrations are more difficult to separate than a scenario where a single material has much greater concentration than the other. As such, the material concentrations in this study were chosen to produce similar attenuation levels between pairs of materials to simulate more difficult separability scenarios. In general, in the presence of k-edge enhanced signals, such separation is more obvious with a threshold selection that emphasizes the difference between those signals. As shown in the separability comparison of lanthanide materials, pairs of materials that had a smaller k-edge difference were more difficult to separate, while the highest separability values were between lanthanide pairs that had a larger separation between their k-edge values. While determination of specific limits of separability for different clinical systems is a task for future studies, the results in this study can be used to inform which pairs of materials can be considered for multi-material contrast tasks.

The materials chosen in this study inclulded agents that are used or are under investigation for clinical applications. Both gadolinium^44^ and bismuth^29^ have been investigated as dual contrast agents to be deployed along with iodine. Multi-contrast CT imaging offers two potential clinical benefits. First, contrast agents can be administered in different ways to directly image two areas as accomplished in studies by Ren et al.^29^ who administered a bismuth solution in the gastrointestinal tract followed by intravenous iodine. Second, by delaying the time of injection between two contrast agents, it becomes possible to image multiple phases with a single acquisition. Symons et al.^24^ administered gadolinium followed by iodine which allowed imaging of arterial and delayed renal enhancement and distinction of both from bismuth administered orally. In an occlusion-reperfusion model of myocardial infarction, Symons et al.^23^ injected gadolinium followed later by iodine allowing imaging of early and late myocardial perfusion. Dual-phase imaging can reduce the overall radiation dose for multi-phase scanning by reducing the overall number of scans required. Nanomaterials offer a different opportunity to extend the application of clinical CT. As nanoparticles can be tagged to different molecules of interest^32^, they can be used for targeted imaging of different regions and functions thus enabling functional imaging with CT.

The lanthanides, outside of gadolinium and ytterbium, are not being widely considered for usage in clinical CT due to potential biocompatibility issues, including toxicity. There are potential uses of these materials for pre-clinical science such as simultaneous imaging of multiple agents or drug development with embolic beads. However, the primary reason for their inclusion was their convenient k-edge range (47–63 keV) which falls in the range of the most commonly used energy thresholds in photon-counting CT. As a result, the lanthanides represent a set of materials that may be used to answer more fundamental questions regarding the separability of materials and the relationship between material k-edges and energy thresholds.

While the development of new contrast agents and nanomaterials offer exciting opportunities to expand the capabilities of diagnostic imaging, there are some considerations before eventual clinical adoption. Many current spectral CT systems, including the one modeled in this study, use two energy channels for their primary acquisition. Conventional approaches to material decomposition are limited to two or three material classes with such an acquisition. Future developments in hardware to include additional imaging channels and software with improved artificial intelligence guided material decomposition may address this challenge. This work represents an important characterization along this trajectory as in silico methods can help define design parameters for future systems and provide data for the development of new algorithms and applications. While we characterized a current clinical system, the statistical methodology used in this work can provide an approach for determining how to best image contrast agents with future systems.

There were some limitations to this study. First, the material concentrations used in this study were chosen to produce similar attenuation to the clinical standard for iodine. In practice, non-iodine materials may need different concentrations in the clinic to account for concerns of toxicity and signal strength. Second, only one spectral CT system was modeled. The sensitivity of separation between materials may vary due to the specifics of different spectral CT scanners as they become available for clinical use and how they achieve spectral separation. For other and future systems, particularly those with more energy bins than the system modeled in this study, an evaluation like our study would have to be performed. Third, the energy spectrum we used was at 120 kV. A higher kV of 140 will generate different but likely similar trends to those seen in this work. Finally, the current study modeled only two energy thresholds. Incorporation of additional energy levels would enable multi-material acquisitions beyond those in this study and is a topic for future evaluation.

## Conclusion

With the development of spectral CT, material identification and quantification across different energy levels has become possible. This has led to an increased interest in materials and nanomaterials that can function as contrast agents, alone or with the clinical standard material of iodine where the two materials may be distinctly identified. In this study, we examined the signal properties and separability of those signals for several candidate materials with varying energy threshold. The impact of the k-edge energy levels on energy threshold selection was shown. Signal was maximized by the selection of a threshold that fell just above the k-edge of materials. For pairs of materials, the threshold value that separated the k-edge signals the best was shown to maximize separability, with specific quantitative results for specific material pairs.

## Methods and materials

### CT simulation pipeline

Simulations in this study were generated using the DukeSim (Duke University, USA) CT simulation platform^43^. The simulator enables realistic simulation of CT acquisitions by incorporating scanner-specific geometry, physics and protocol conditions^58^ and has been validated for both conventional and photon-counting CT systems^59^. The photon-counting CT detector model was provided by the vendor and includes modeling of cross-talk and pulse pileup corrections.

The simulation pipeline requires information on the object to be imaged, the material properties of that object, and details of the imaging system to be modeled. Computational phantoms are used to represent the patient or object. Scanner properties include hardware information, such as detector and x-ray source conditions, as well as acquisition parameters, such as tube rotation speed and x-ray tube conditions. Two modules are used to carry out the simulation. First, a ray-tracing module is used to generate the primary signal at the detector. Second, a Monte Carlo module^60^ is used to estimate scatter signal and dose. The outputs from both modules are post-processed for additional steps including tube current modulation^58^, noise addition, and air/water corrections. The final outputs are generated as projection images. Images were reconstructed using the Multi-Channel Reconstruction^61^ toolkit.

### Simulation design and image acquisition protocol

In this study, a clinical photon-counting CT system^62^ (NAEOTOM Alpha, Siemens Healthineers, Forchheim, Germany) was modeled operating in QPlus mode which uses two energy thresholds, a 144 × 0.4 mm collimation, and 0.4 × 0.4 mm in-plane pixels. Acquisitions were done with a fixed tube voltage (120 kV) and dose (tube current − 313 mAs, CTDI_vol_ − 24.0 mGy). Images were simulated with a fixed low threshold at 20 keV while the higher energy threshold was varied between 50 and 90 keV in steps of 5 keV. The resulting output was two images for each acquisition condition: a low energy image containing signal from photons between the two thresholds and a high energy image containing signal between the high threshold and the maximum value of 120 kV. Images were reconstructed with filtered-back projection and 0.5 × 0.5 mm pixels. Images were denoised using 3-D Gaussian filtering (*imgaussfilt3* function, MATLAB, v2021a, MathWorks, Natick, MA) before further processing.

### Image quality evaluation

In each image, circular regions of interest (ROI) were drawn in each vial and voxel values extracted for further analysis. An additional circular ROI was drawn in the water background. Image quality was evaluated as noise for each image and contrast between each material insert. Noise was characterized by the standard deviation of voxel intensities within the water background ROI. Contrast between each material and background was calculated by

1 $$\:Contrast={\mu\:}_{insert}-{\mu\:}_{background}$$

where $\:{\mu\:}_{i}$ represents the mean value within each ROI. Contrast was calculated separately for both low and high energy images.

### Separability evaluation

To evaluate the separability between materials, a separability index^53^ was used to compare each material at every energy threshold level. For two given regions of interest, this method calculates the expected overlap in signal component. The distribution of intensities within each multi-energy ROI is modeled as a multivariate random process with a deterministic signal and a stochastic noise component. Each variate represents a different energy level in the original spectral CT acquisition. The linear Hotelling observer^63^ is used to calculate a test statistic $\:\lambda\:$ for an ROI of interest. The test statistic represents the probability that a randomly chosen voxel value belongs to a particular material and the calculation of the test statistic accounts for factors that influence signal quality including material concentration. For two materials A and B, the separability index is defined as

2 $$\:{s}^{{\prime\:}}=\frac{\stackrel{-}{{\lambda\:}_{A}}-\stackrel{-}{{\lambda\:}_{B}}}{\sqrt{\left(\frac{1}{2}\right)\left({\sigma\:}_{{\lambda\:}_{A}}^{2}+{\sigma\:}_{{\lambda\:}_{B}}^{2}\right)}}$$

where $\:\stackrel{-}{\lambda\:}$ and $\:{\sigma\:}_{\lambda\:}^{2}$ represent the mean and variance for each test statistic.

## Data Availability

The data used to support the findings of this study are available from the corresponding author upon request.
